# Tailored modern GERD therapy – steps towards the development of an aid to guide personalized anti-reflux surgery

**DOI:** 10.1038/s41598-019-55510-2

**Published:** 2019-12-16

**Authors:** Milena Nikolic, Katrin Schwameis, Matthias Paireder, Ivan Kristo, Georg Semmler, Lorenz Semmler, Ariane Steindl, Berta O. Mosleh, Sebastian F. Schoppmann

**Affiliations:** Department of Surgery, Division of General Surgery, Medical University of Vienna, Waehringer Guertel 18-20 1090 Vienna, Austria

**Keywords:** Cancer prevention, Gastro-oesophageal reflux disease, Outcomes research

## Abstract

As the incidence of gastroesophageal reflux disease (GERD) is rising, surgical treatment is continuously advancing in an effort to minimize side effects, whilst maintaining efficacy. From a database of patients that underwent anti-reflux surgery at our institution between 2015 and 2018, the last 25 consecutive patients that underwent electrical stimulation (ES), magnetic sphincter augmentation (MSA) and Nissen fundoplication (NF), following a personalized treatment decision aid, were included in a comparative analysis. After preoperative evaluation each patient was referred for an ES, MSA or NF based on esophageal motility, hiatal hernia (HH) size and the patients’ preferences. Postoperative gastrointestinal symptoms and GERD-Health-related-Quality-of-Life were assessed. Preoperatively the median DCI (299 ES vs. 1523.5 MSA vs. 1132 NF, p = 0.001), HH size (0.5 cm ES vs. 1 cm MSA vs. 2 cm NF, p = 0.001) and presence of GERD-related symptoms differed significantly between the groups. The highest rate of postoperative dysphagia was seen after MSA (24%, p = 0.04), while the median GERD HRQL total score was equally distributed between the groups. The positive short-term postoperative outcome and patient satisfaction indicate that such an aid in treatment indication, based on esophageal motility, HH size and patient preference, represents a feasible tool for an ideal choice of operation and an individualized therapy approach.

## Introduction

Gastroesophageal reflux disease (GERD) is a public health issue, affecting up to 33% of the population worldwide, spanning across all age groups and both sexes^1,2^. In the United States alone, this common disease produces a financial burden of $9 to $10 billion per year in direct costs, largely due to the use of proton pump inhibitors as its first line treatment^2^. Although 40% of GERD patients remain symptomatic under medical treatment and proton pump inhibitors have been associated with various long-term adverse effects, rates of anti-reflux surgery have significantly decreased since achieving a peak in 2009^3–8^. The main reasons why more physicians are restrictive when undertaking surgical GERD-treatment and less patients opt for it are long term side effects such as persistent dysphagia and gas-bloat syndrome^4,5,8,9^.

Since the second half of the 20^th^ century the gold standard in GERD therapy – Nissen fundoplication (NF), underwent countless modifications in an effort to maximize its efficiency while reducing the side-effect rate^10,11^. The laparoscopic 360-degree fundoplication has been tailored to a 270-degree fundoplication (Toupet) as well as an anterior 120-degree fundoplication (Dor)^11,12^. Although both modifications have been reported with lower dysphagia rates NF remains superior in long-term reflux cessation and symptom relief^3,12–15^. In an effort to further minimize this therapy gap in GERD treatments surgical novelties such as magnetic sphincter augmentation (MSA) and electrical stimulation (ES) of the lower esophageal sphincter (LES) are becoming more prevalent^2,16–24^. By mechanically enhancing the LES, while preserving the hiatal anatomy, MSA has been proven safe and comparably effective to NF with a lower rate of side effects^9,18,25,26^. Although no case-controlled analysis of ES versus NF exists to date, long-term 3 year results of ES show a reduction in GERD symptoms as well as improvements in esophageal acid exposure with no new gastrointestinal side effects^2,20–24^. Nevertheless, the lack of longer term studies (>5 years) of safety, as well as efficacy in comparison to the gold standard, still prevent the implementation of these novel therapies into the guidelines in GERD therapy. In a commencing time of optimizing and personalizing anti-reflux surgery, where still further research is needed in order to recommend the widespread use of MSA or ES, we aimed to develop a tool, to help in the decision process of indicating one of the distinct anti-reflux operations and consequently result in positive postoperative outcomes^27,28^.

Aim of this study was to analyze the short-term postoperative symptom control, adverse effects, and patient satisfaction in patients undergone laparoscopic ES, MSA and NF respectively, according to an established treatment decision aid and subsequently the safety and feasibility of such in a high output specialized reflux center.

## Methods

### Preoperative assessment

All patients received a standardized interview, clinical examination, an upper GI endoscopy, a video esophagram and esophageal functioning tests consistent of a high-resolution manometry and a 24-hour-Impedance-pH-metry. GERD was diagnosed by positive pH results or increased total reflux episodes with positive symptom correlation on esophageal functioning tests, presence of esophagitis on endoscopy or typical GERD symptoms sensitive to PPI medication. Hiatal hernias were diagnosed with high precision using both upper GI endoscopy and high-resolution manometry. Ineffective esophageal motility (IEM) is classified in line with the updated Chicago Classification v3.0 calculating the distal contractile integral (DCI) on high-resolution manometry^29^. The DCI represents an index of contractile vigor calculated as the product of amplitude, duration, and span of the distal esophageal contraction and should have a range of 500–50000 mmHg-cm-s. A total of 10 swallows are assessed; if 50% or more have a DCI < 500 mmHg-cm-s an IEM is diagnosed.

### Development of the treatment decision aid

**Figure 1:**
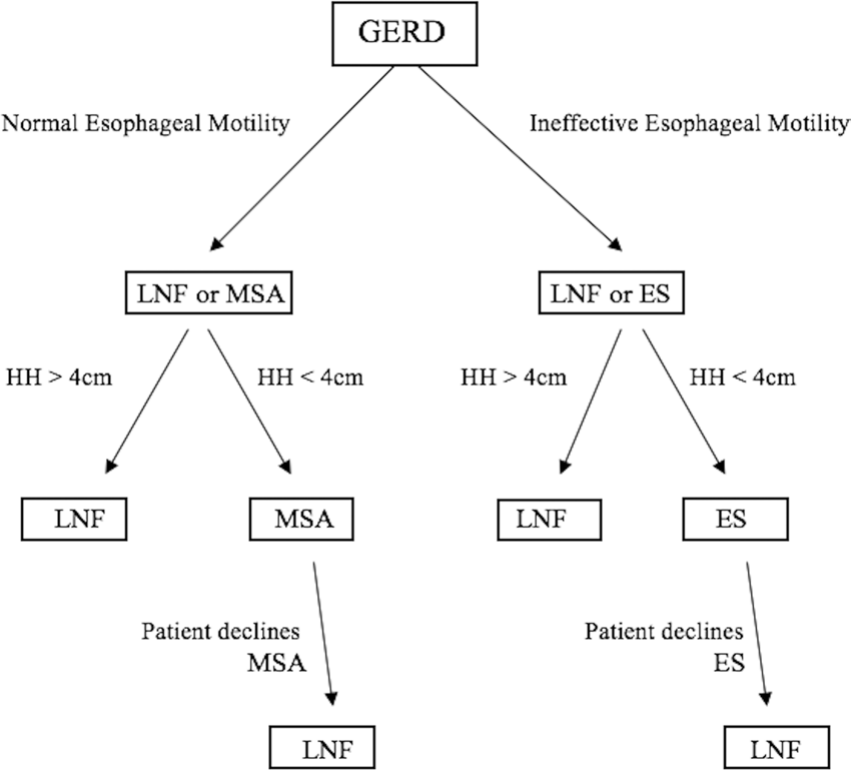
Personalized anti-reflux treatment decision aid.

All patients were primarily divided into two groups according to IEM. Patients with normal esophageal motility were then assessed based on hiatal hernia size: those with a hiatal hernia larger than 4 cm underwent NF, while those with a hernia smaller than 4 cm received either MSA or NF in case the patient refused MSA. Patients with ineffective motility were then also assessed based on hiatal hernia size: those with a hernia larger than 4 cm underwent NF, while those with a hernia smaller than 4 cm underwent ES or NF in case the patient refused ES. Patients were thoroughly informed about the risks and benefits of each individual surgery method as well as the fact that current guidelines can only recommend the standard therapy – the laparoscopic fundoplication. A graphic depiction of our personalized GERD diagnostics and anti-reflux surgery is shown in Fig. 1. From a database, all symptomatic GERD patients that had undergone ES, MSA or NF since all three surgical options were available at our institution, between 2015 and 2018 were identified. Information about the preoperative evaluation, surgical procedure and postoperative outcome was obtained. For better comparison, additionally, the last 25 consecutive patients that had undergone ES, MSA and NF in our institution were included in a direct compared analysis (n = 75).

### Surgery

All procedures were performed by the same specialized upper gastrointestinal surgical team. The surgical approach was laparoscopic in all cases. All procedures were standardized regarding the surgeon’s and patient’s positions (anti-Trendelenburg), trocar sites and instruments used. A hiatal closure was performed in all NF cases, and in the MSA and ES cases only if a hiatal hernia was present. These procedures were conducted by hiatal dissections and crural closures with 2–5 stitches using non-absorbable sutures. All cases were performed without the use of an esophageal bougie.

### Electrical Stimulation of the LES

ES was performed as further described: The ES system contains a bipolar stimulation lead with two stitch electrodes, an implantable pulse generator (IPG), and an external programmer. The IPG is connected to the stimulation leads and implanted in the left lower quadrant of the abdomen subcutaneously, while it’s evaluation and programing is provided via a wireless external programmer and computer software. Until September 2017 all patients were programmed to receive 12 stimulations for 30 minutes in 24 h, whereas from September 2017 there was a change of regime to 16 stimulations for 20 minutes in 24 h.

The surgical procedure itself starts with a brief mobilization of the anterior aspect of the intraabdominal esophagus. The electrodes are placed in the muscularis propria in the abdominal esophagus, and in an inline position, approximately 10 mm distal to the first electrode at the gastroesophageal junction. This is performed under endoscopic visualization to rule out perforation into the esophageal lumen. The stimulation lead connector is then attached to the implantable pulse generator. Finally, a functionality test is performed by the technical support personnel.

### Magnetic sphincter augmentation

MSA was performed as previously described^30^: briefly after the mobilization of the esophagogastric junction, the adequate ring size was measured with the sizing tool and the magnetic ring was wrapped around the lower end of the lower esophageal sphincter.

### Nissen fundoplication

NF was performed in a highly standardized technique as recently explained^31^. In brief: both crus of the diaphragm were dissected using the ultrasonic dissector in order to expose the distal esophagus. Special care was taken to achieve an adequate “intraabdominalisation” of the lower esophagus of at least 3 cm in length. An extra-short warp, measuring a maximum of 1.5 cm with the naked eye was created using 2 close stiches with non-absorbable sutures. Division of the small gastric vessels was avoided; however special care was taken to complete mobilization of the fundal adhesions to the diaphragm. The first stich included the anterior esophageal wall. The vagal nerve was always identified and included in the wrap. After the surgery was completed, a blunt laparoscopic instrument was placed between the posterior esophageal wall and the wrap in order to determine the looseness of the fundoplication.

### Postoperative care

Postoperative, all patients undergone NF received a restricted semiliquid food diet for the first 10 days, slowly progressing to solid food in order to avoid dysphagia during the development of the mucosal edema. On the contrary, patients who underwent MSA received an unrestricted diet, putting an emphasis on regular intake of foods every two hours, to avoid the development of dysphagia due to formation of scar tissue surrounding the device. After at least one overnight stay, patients were discharged from the hospital once they were showing an unremarkable postoperative contrast swallow.

On the first postoperative day a contrast swallow with diatrizoate was performed in all patients.

### Postoperative assessment

Due to distinct preoperative characteristics, such as esophageal motility and hiatal hernia size, patients were not case-matched. The last and consecutive 25 patients that underwent NF, ES and MSA in our specialized center were included in a comparative analysis in order to minimize the selection bias.

The median follow-up time was 3 months. Short-term follow-up was performed by the same physician using a standardized interview that assessed postoperative gastrointestinal symptoms, proton pump inhibitor intake and GERD-Health-related-Quality-of-Life score (GERD-HRQL). The frequency and severity of postoperative dysphagia was assessed using the classification of Saeed *et al*., where the ability to swallow can be scored from 0 to 5, where 0 implies the inability to swallow and 5 indicates normal swallowing^32^.

Adverse effects such as complications, hospital readmission, emergency surgery or elective re-operation were documented. Patients with recurrent symptoms received upper GI endoscopy as well as esophageal functioning tests.

### Statistical analysis

The Dataset used includes a Table of preoperative patient characteristics (sex, age, BMI, esophageal functioning tests findings, GERD-related symptoms), intraoperative and perioperative data and an early postoperative follow-up including total GERD HRQL score, postoperative dysphagia, intervention, complication and revision rates.

Statistical analysis was performed using SPSS® statistics 20.0 (IBM, Armonk, NY). The data was described using median (interquartile range) or mean (range). Statistical analysis appropriate for non-parametric data was used. Categorical variables were assessed using the Fisher exact test and continuous data using the Wilcoxon Rank test as appropriate. Statistical significance was defined as a *p*-value < 0.05.

This study (2293/2017) was approved by the Institutional Review Board of the Medical University of Vienna, Austria. Methods were carried out in accordance with relevant guidelines and regulations. Individual informed consent was not acquired, due to retrospective study design and national regulations stating that individual informed consent is not required when the data is obtained from a secure patient database and no interaction with the patient himself is needed.

## Results

A total of two hundred and sixty-seven (n = 267, NF n = 169 (63%), MSA n = 73 (27%) and ES n = 25, (11%)) patients underwent laparoscopic anti-reflux surgery in a period of three years. Demographics and preoperative findings of all GERD patients are summarized in Table 1.

**Table 1:** Demographic data and results of preoperative diagnostics of all GERD patients.

	ES	MSA	NF
Total n = 267 (100%)	N = 25 (8%)	N = 73(24%)	N = 169 (68%)
Sex (M vs. F)	11 vs. 14	52 vs. 21	110 vs. 97
Median Age (IQR)	54	49	54
Median BMI (IQR)	25	25	27
Presence of HH	N = 16	N = 62	N = 164
Median size of HH(cm)	0.5	2	3
Median Total pH < 4%	9.6	7.9	7.9
Median Total Reflux episodes	79	64	67
Median LES EE pressure	16.5	20	18.45
Median IRP	10	10	10
Median DCI	299	1626.5	1102
Ineffective Motility	15	0	22

In this study seventy-five (n = 75, 42 male and 33 female) consecutive patients were further included in a comparative analysis study and divided into three groups: 25 ES, 25 MSA and 25 NF. There was no significant difference in the median age or preoperative BMI between the groups. A hiatal hernia was present in sixty-six of the patients, two from which were type III hernias using the classification of Hill *et al*.^33^ A difference in median hiatal hernia size was found between the groups.

The GERD-associated preoperative symptoms were divided in typical (heartburn and/or regurgitation), atypical (hoarseness and/or cough) and combined. While typical GERD symptoms differed significantly between the three groups, atypical GERD symptoms and combined GERD symptoms were equally distributed between the ES-, MSA- and NF-group. Furthermore, no difference was seen in preoperative dysphagia-rates in patient that underwent ES, MSA and NF respectively. Demographics and preoperative findings of the 75 consecutive patients are shown in Table 2.

**Table 2:** Demographic data and results of preoperative diagnostics of 75 consecutive patients.

	ES	MSA	NF	
Total n = 75 (100%)	N = 25 (33.3%)	N = 25 (33.3%)	N = 25 (33.3%)	
Sex (M vs. F)	11 vs. 14	16 vs. 9	15 vs. 10	
Median Age (IQR)	54	56	55	p = 0.686
Median BMI (IQR)	25	26	26	p = 0.896
Presence of HH	N = 16	N = 21	N = 25	p = 0.039
Median size of HH(cm)	0.5	1	2	p = 0.001
Presence of BE	3	1	2	p = 0.522
**Symptoms:**				
Typical GERD	N = 13	N = 18	N = 9	p = 0.038
Atypical GERD	N = 8	N = 4	N = 2	p = 0.08
Combined	N = 4	N = 3	N = 3	p = 0.891
Dysphagia	N = 3	N = 3	N = 1	p = 0.532

**Figure 2:**
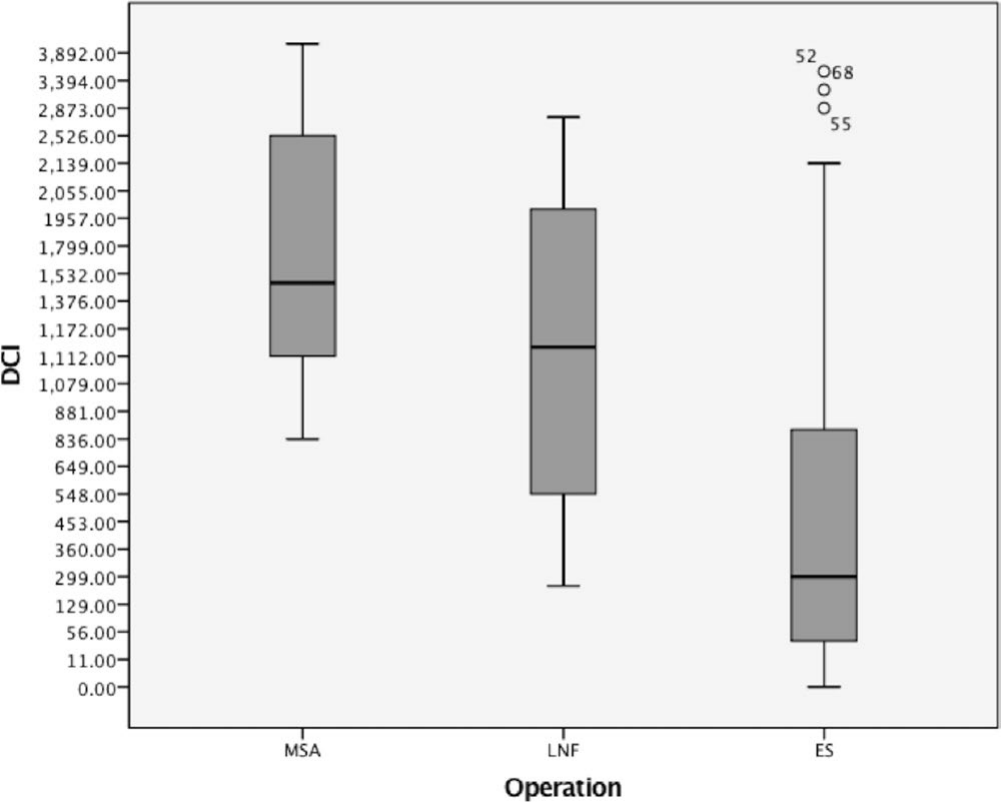
Preoperative DCI in patients undergoing ES, MSA and NF.

The data from the preoperative esophageal functioning tests (EFTs) was available from seventy-three (n = 73, 97%) of the patients. The predicted difference in the mean distal contractile integral (DCI) is graphically portrayed in Fig. 2. Accordingly, the rate of ineffective esophageal motility prior to surgery showed a significant difference in the distribution between the groups. When further comparing the median total pH < 4%, total number of reflux episodes, lower esophageal sphincter end expiratory (LES EE) pressure and integrated relaxation pressure (IRP), we found no difference between the groups. Detailed comparison of preoperative esophageal functioning tests, including the median total pH < 4%, total reflux episodes, LES EE pressure, IRP, DCI and ineffective motility is shown in Table 3.

**Table 3:** Results of preoperative esophageal functioning tests.

	ES	MSA	NF	
Median Total pH < 4%	9.6	6.6	7.3	p = 0.139
Median Total Reflux episodes	79	57	63	p = 0.527
Median LES EE pressure	16.5	20	22.9	p = 0.348
Median IRP	10	11	10	p = 0.493
Median DCI	299	1523.5	1132	p = 0.001
Ineffective motility	15	0	6	p = 0.000

### Outcome

#### Operative parameters

There was a significant difference in the median operation time between the three anti-reflux surgery methods. All cases were performed laparoscopically without conversions to open surgery. Patients undergoing NF had the highest rate of additional posterior crural closure, followed by MSA and ES, respectively. There was no blood loss observed and no intraoperative complications occurred in any of the surgeries. Furthermore, the median length of stay (LOS) was significantly different between the three groups with the shortest LOS in patients after MSA, followed by NF and ES, respectively. The intraoperative data is demonstrated in Table 4.

**Table 4:** Intraoperative and perioperative data.

	ES	MSA	NF	
Median OR duration (min)	58	30	60	p = 0.000
Hiatal repair	N = 14	N = 21	N = 25	p = 0.000
Median blood loss (ml)	0	0	0	—
Intraoperative complications	0	0	0	—
Median length of stay (days)	3	2	3	p = 0.025

#### Symptom relief

We observed no difference in the postoperative median total GERD HRQL-scores (4 (ES) vs. 2.5 (MSA) vs. 3 (NF), p = 0.066).

#### Side-effects

In the short term postoperative follow-up dysphagia, defined as ≤3 using the classification of Saeed *et al*., was reported by six (n = 6/25, 24%) patients that underwent MSA, four (n = 4/25, 16%) patients that underwent NF and by none (n = 0/25, 0%) of the patients that underwent ES, with a significant difference between them. All of the patients that underwent MSA and three of the patients that underwent NF, that reported postoperative dysphagia, defined it as an occasional difficulty swallowing solids. One patient, who had undergone NF developed the inability to swallow solids and liquids. The postoperative contrast swallow showed delayed passage of the contrast in the gastroesophageal junction, while the esophagogastroduodenoscopy showed no stenosis and a regular position of the Nissen wrap. The patient then underwent two further endoscopic balloon dilatations, with temporary, short-term relief and reoccurrence of the dysphagia. One patient, who had undergone MSA developed postoperative delayed gastric emptying and underwent Botox injection of the pylorus. No patients underwent revision surgeries and no postoperative complications were observed in the short term follow-up. Postoperative follow-up results are displayed in Table 5.

**Table 5:** Early postoperative median follow-up of 3 months.

	ES	MSA	NF	p-Value
Median total HRQL-score	4	2.5	3	p = 0.066
Dysphagia*	0	6	4	p = 0.04
Endoscopic intervention	0	1	1	p = 0.598
Revision surgery	0	0	0	—
Postoperative complication	0	0	0	—

*Dysphagia was defined as ≤3 using the classification of Saeed *et al*.^32^.

## Discussion

From the novelty of the NF in 1991 to the wide-spread use of a titanium ring as a sphincter augmentation device, as well as an “esophageal pacemaker” intended to aid in the physiological sphincter contraction, history has shown, that the “one perfect operation” does not exist^10^. Based on preoperative esophageal functioning tests, disease severity determined by endoscopy, detailed patient history and GERD-related symptom presentation, each individual patient would benefit from one surgical therapy more than another.

The aim of our study was to show feasibility and safety of a developing treatment decision aid based on short-term postoperative outcomes in patients that underwent NF, MSA and ES in our high input reflux-center. Up to date, no guidelines include MSA or ES in anti-reflux surgical therapy and no study has been published with such a comparison between these three anti-reflux surgical treatments.

Our treatment decision aid was assessed based on previous studies, where ineffective esophageal motility was a contraindication for MSA and hiatal hernias bigger than 3 cm were a contraindication for both MSA and electrical sphincter stimulation^16,23,26^. As Rona *et al*. showed that MSA can be safely and effectively performed in hiatal hernias up to 7 cm in size with additional crural closure, our cutoff point was increased to ≥4 cm as an exclusion criteria for MSA and ES. Lastly patients with hiatal hernias ≤ 4 cm and IEM had the possibility of choosing between the NF and ES, while patients with hiatal hernias ≤ 4 cm and normal motility had the possibility of choosing between the NF and MSA.

When looking at the results of the preoperative EFTs in our study we observed no statistical significant difference in the median total pH < 4%, median total reflux episodes, median LES EE pressure or median IRP between the patients undergoing these three distinctive anti-reflux surgeries. Due to our treatment decision aid based primarily on the esophageal motility, the only, but expected significant difference in the preoperative EFTs between the groups was the median DCI. The reason why the assessment of the preoperative esophageal motility based on the EFTs was our most important factor for indicating the type of surgical GERD-treatment can be explained knowing that the most feared complication of anti-reflux surgery is dysphagia, persisting beyond 8 weeks postoperatively. Still not fully understood, possible risk factors for its development are believed to be a tight hiatus or slipped fundoplication, as well as preoperative esophageal motility disorders^13,34,35^. Hence, the first step in avoiding this adverse effect lies in the correct preoperative diagnostics and choice of operative management.

Patients with ineffective esophageal motility either underwent NF or ES, whereas patients with normal motility underwent either NF or MSA (Fig. 1). In our study we observed six patients (n = 6/25, 24%) with ineffective esophageal motility, who underwent NF. In the direct postoperative median follow-up of 3 months one patient (n = 1/25, 4%) developed persistent dysphagia, requiring multiple endoscopic dilatations. This rate of persistent dysphagia is on the lower end to previously published literature, ranging from 2.2% in a retrospective analysis of 350 patients after NF in our institution to 11% in a prospective European study comparing medical to surgical treatment of GERD^31,36,37^. Investigating postoperative dysphagia in this collective, MSA patients had a higher rate of short-term postoperative dysphagia than patients undergone NF and ES (24% vs. 16% vs. 0%, p = 0.04). As the magnetic ring is implanted around the weak esophageal sphincter, patients need time to adjust to the foreign body. Moreover, they are immediately started on a strict solid food diet to prevent fibroses, so higher rates of early postoperative dysphagia can be seen after MSA rather than NF. We observed no complications or new undesirable effects in patients that undergone ES, confirming previous literature and supporting it as the potential ideal therapy for GERD with an incomparable “magnitude of effect and lack of side effects”^38^.

When comparing the median hiatal hernia size on endoscopy and high-resolution manometry preoperatively, we can see a statistically significant difference between the three groups of patients. Hiatal hernia size was the second deciding factor in our choice of anti-reflux surgery (Fig. 1) as a bigger hernia requires more extensive dissection thus making the surgery more invasive and increasing the risk of possible adverse effects. Our results confirm previous findings as MSA and ES were primarily contraindicated in patients with a hiatal hernia size larger than 3 cm. Nevertheless, as mentioned above Rona *et al*. published the first positive MSA results where an additional hiatoplasty was performed in hiatal hernia sizes up to 7 cm^26^. Following these footsteps we increased our cutoff to ≥4 cm as an exclusion criteria for MSA and ES.

As the main goal of GERD treatment is symptom relief, increase in quality of life and prevention of long-term disease side-effects our last criteria in choice of GERD treatment was patient’s preference of surgery (Fig. 1). As the most feared side-effect of anti-reflux surgery is dysphagia, patients with typical GERD symptoms or proton pump inhibitor-sensitive GERD usually prefer a less invasive method of treatment than NF. When comparing the preoperative GERD-related symptoms we can see a significant difference in the number of patients with typical GERD symptoms between the three groups. As previous research has shown that patients with typical symptoms usually show better postoperative outcome than their counterparts, it is not surprising to see the patient group undergoing the most radical operation showing the least number of typical GERD-related symptoms^39,40^.

At a median follow-up of 3 months we found no difference between the three groups when comparing the postoperative quality of life and patients’ satisfaction based on the mean Total GERD-HRQL score. Studies so far have compared MSA and NF, also finding no difference in postoperative symptom-relief or patient satisfaction. Although desirable, no controlled studies between ES and NF or MSA exist to date^9,18,25^.

As minimally invasive surgery developed, the main advantages were thought to be the smaller incisions and shorter hospital stay. As the learning curve progressed, the durations of the operations decreased. Compared between ES, MSA and NF we can see that MSA lasts significantly shorter than ES and NF. This can be explained with the nature of the surgical procedure, as during the MSA minimal hiatal dissection and fundus mobilization is performed, in contrast to a NF. Furthermore, during an ES additional EGD needs to be performed to control for mucosal damage and the device function has to be tested. Likewise, in concordance with previous literature the LOS was shorter in patients undergoing MSA compared to NF^25^.

None of our patients during the short term follow-up required revision surgery. We observed one patient needing two endoscopic dilatations after NF due to postoperative dysphagia, without any surgical or anatomical correlate. Previous literature also found no differences in postoperative complications between MSA and NF. ES has not yet been clinically compared to either method^9,18,25^.

Neither MSA nor ES are meant to replace NF, but rather provide a less invasive surgical alternative to patients that have not yet progressed to severe GERD complications, show only partial response to proton pump inhibitors and are hesitant to undergo a more radical solution. Advances in GERD treatment don’t come with a “one size fits all” approach but rather with personalized reflux therapy, aimed at choosing the single best operation, based on detailed preoperative diagnostics and patient symptoms.

The major limitations of our study are its retrospective nature, lack of a control group and short-term follow-up. As MSA and ES are still novel surgical techniques and thus not a part of official GERD guidelines, a direct, controlled, comparison between our treatment decision aid and current guidelines was difficult as such. Although multiple case control studies with MSA and NF have been published, case-controlled studies of ES versus NF as well as long term studies of more than 5 years are necessary. Additionally, as the main factor in the preoperative EFTs influencing the type of GERD therapy was the esophageal motility and hiatal hernia size, thus also being the main difference in the patient characteristics, a comparison in postoperative outcome is consequently biased. A randomized and controlled clinical trial is the next needed step in order to show the efficacy and long term safety of such a decision aid in innovative anti-reflux treatments.

## Conclusion

The main differences and the deciding factors in our aid for choice of GERD therapy were found to be the preoperative DCI and subsequently the presence of ineffective esophageal motility, hiatal hernia size and the patient’s preference. The overall low postoperative dysphagia-rate and no significant differences in symptom control and patient satisfaction rates between the three surgical treatments show that such a treatment decision aid is feasible in the short-term postoperative time and could be considered in surgical anti-reflux evaluation.

## Data Availability

The datasets generated and analysed during the current study are available from the corresponding author on reasonable request.
